# Supplementary material for: Structure of ATTRv-F64S fibrils isolated from skin tissue of a living patient
Source: Nat Commun. 2025 Dec 16;17:781. doi: 10.1038/s41467-025-67457-2 (PMC12824241; doi:10.1038/s41467-025-67457-2)
Supplement: Supplementary file 1 — Supplementary Information [file 41467_2025_67457_MOESM1_ESM.docx]

**Supplementary Figure 1: Characterization and extraction of ATTR fibrils from skin tissue. a**, Amyloid deposits in skin biopsies stained with Congo red. Top, 50 μm thin sections were examined under bright-field (BF) microscopy. Magnification 4x, scale bar 200 μm. Middle, Congo red deposits with characteristic birefringence under polarized light (PL) are shown from the inserts (dashed rectangles). Magnification 10x, scale bar 200 μm. Bottom, Representative immunofluorescence images showing a reduced intraepidermal innervation in ankle and thigh tissue. Thin sections were stained with anti-PGP9.5 antibody (green) and the nuclear stain DAPI (blue). Sections were analysed with an inverted fluorescence microscope. PGP9.5-positive fibres crossing the dermal-epidermal junction (dotted line) were counted according to published protocols^1^. Magnification 40x, scale bar 50 μm. **b,** Silver stained SDS-PAGE gel of samples from washing and extraction steps during amyloid fibril extraction from an ATTRv-F64S patient. Fibrils were extracted from 5–10 mg of ankle and thigh skin tissues. The TTR protein is indicated by a dashed box. **c**, Negative staining micrographs of ATTRv-F64S fibrils from ankle and thigh tissues. A minimum of five micrographs were collected for each tissue. Scale bar, 500 Å.

**Supplementary Figure 2: LC-MS/MS analysis of ATTR fibrils extracted from skin tissue. a-b,** Alignment of the liquid chromatography–tandem mass spectrometry (LC-MS/MS) identified peptides in fibrils extracted from ankle (**a**) or thigh (**b**) biopsies with the sequence of mature transthyretin, yielding a 92.9% coverage in both sites. F64S mutation is indicated in red. **c** and **d**, Analysis of the N-terminus of transthyretin using an N-free semi-specific search of the LC-MS/MS data. The alignment shows an increased total coverage of the mature form of the transthyretin (TTR) protein to 98.4% in fibrils extracted from the ankle biopsy (**c**), and to 93.7% in fibrils extracted from thigh biopsy (**d**).

**Supplementary Figure 3: LC-MS/MS analysis of a bulk skin biopsy sample from ankle. a**, Annotated MS/MS spectra of the wild-type (top) and F64S-mutant (bottom) peptides in bulk skin fibrils. **b**, Extracted LC-MS ion chromatogram of the wild-type (dark green) and F64S-mutant (pink) peptides. **c**, TIC-normalised peak areas show similar intensity levels between wild-type and mutant peptides. Source data are provided as a Source Data file.

**Supplementary Figure 4: Cryo-EM analysis of ATTRv-F64S fibrils. a**, Representative two-dimensional (2D) class averages of single twisted protofilaments extracted from ankle skin. Scale bar, 100 Å. **b**, Representative two-dimensional (2D) class averages of twist-dimer ATTRv-F64S fibrils from ankle skin. Scale bar, 100 Å. **c**, Gold standard Fourier Shell Correlation (FSC) curve of the cryo-EM map. The FSC curve between the cryo-EM map and the atomic coordinates was calculated using Mtriage^2^. **d**, Local resolution map of ATTRv-F64S fibril color-coded according to the local resolution ranging from 2.6 to 3.8 Å. **e**, The F64S mutation likely destabilizes the TTR protein. Left, Structure of the TTR tetramer shown as a cartoon (PDB ID: 4TLT^3^). Middle, close-up view of hydrophobic interactions between F64 and surrounding residues (distance < 4 Å) in a single monomer. Right, Energy differences between wild-type TTR protein and four F64-related mutants that have been identified in patients, which were calculated using FoldX^4^. Source data are provided as a Source Data file.

**Supplementary Figure 5: PTM sites identified by MS mapped on the tetrameric and fibril structures. a**, Post-translational modification (PTM) sites are mapped onto the ATTR-F64S fibril structure. The disordered region (residues 37-56) is shown as a dashed line. T49 and S52 are shown as black dots and all other residues identified are shown as sticks. **b**, PTM sites are mapped onto the structure of the TTR tetramer (PDB ID: 4TLT^3^).

**Supplementary Table 1: Post-translational modifications analysis on LC-MS/MS data of ATTRv-F64S fibrils.**

Sites are filtered for both localization probability of the modification site and spectral similarity to predicted MS/MS spectra above 80%. The PTM site is highlighted in red, while the F64S mutation is shown in green. Reported with * are two sites slightly below the spectral similarity threshold.

**Supplementary Table 2: Cryo-EM data collection, refinement, and validation statistics.**

| **ATTRv-F64S amyloid fibril** | | |
| --- | --- | --- |
| **Data collection** | | |
| Microscope | Titan Krios | |
| Voltage (keV) | 300 | |
| Magnification | 165,000 | |
| Electron dose (e^-^/Å ^-2^) | 40 | |
| Detector | Falcon 4i | |
| Energe filter | Selectris X | |
| Pixel size (Å/pixel) | 0.726 | |
| Defocus range (μm) | -0.6 to -2.0 | |
| Number of micrographs | 9,763 | |
| **Reconstruction** | | |
| Box size (pix) | 280 |  |
| Total extracted segments | 4,599,018 |  |
| Number of segments after 2D classification | 135,574 |  |
| Number of segments after 3D classification | 12,633 |  |
| Symmetry imposed | C1 |  |
| Helical rise (Å) | 4.78 |  |
| Helical twist (°) | -1.36 |  |
| Crossover length (Å) | 633 |  |
| Resolution (global Å)  FSC threshold  Map sharpening B-Factor (Å2) | 2.82  0.143  -20 |  |
| **Model composition** |  |  |
| Protein residues | 855 |  |
| **Refinement** |  |  |
| Resolution (Å) | 2.9 |  |
| FSC threshold  Model to map scores  -CC | 0.5  0.87 |  |
| *B* factors (Å^2^)  Protein residues | 71.02 |  |
| R.m.s deviations |  |  |
| Bond lengths (Å) | 0.008 |  |
| Bong angles (°) | 1.031 |  |
| **Validation** |  |  |
| Clashscore, all atoms | 14.0 |  |
| Rotamer outliers (%) | 0.1 |  |
| **Ramachandran plot** |  |  |
| Favoured (%) | 90.96 |  |
| Allowed (%) | 9.04 |  |
| Outliers (%) | 0.00 |  |
| **Deposition** |  |  |
| PDB ID | 9HYW |  |
| EMDB ID | EMD-52519 |  |

**Supplementary Table 3: Cryo-EM structures of ATTR amyloid fibrils (wild-type and variants).**

| Amyloid fibril | RMSD Ca atoms  (Vs ATTRv-F64S) | Resolution (Å) | PDB ID |
| --- | --- | --- | --- |
| ATTRv-F64S, skin | **-** | 2.8 | 9HYW |
| ATTRwt, heart, patient 1 | 0.633 | 2.8 | 8ADE |
| ATTRwt, heart, patient 2 | 0.672 | 3.3 | 8E7D |
| ATTRwt, heart, patient 3 | 1.242 | 3.3 | 8G9R |
| ATTRwt, heart, patient 4 | 0.893 | 3.3 | 8GBR |
| ATTRwt, heart, patient 5 | 0.594 | 3.4 | 8E7H |
| ATTRv-V20I, heart | 0.806 | 3.4 | 8PKE |
| ATTRv-P24S, heart | 1.198 | 3.7 | 8E7I (unpublished) |
| ATTRv-V30M, heart | 0.649 | 3.0 | 6SDZ |
| ATTRv-V30M, eye | 1.150 | 3.2 | 7OB4 |
| ATTRv-G47E, heart | 0.709 | 2.4 | 8PKF |
| ATTRv-I84S, heart, absent gate | 1.398 | 3.6 | 8E7E |
| ATTRv-I84S, heart, broken gate | 0.911 | 3.1 | 8E7J |
| ATTRv-I84S, heart, closed gate | 0.685 | 3.1 | 8TDN |
| ATTRv-I84S, heart, open gate | 0.899 | 3.1 | 8TDO |
| ATTRv-V122I, heart | 0.668 | 3.0 | 8PKG |

**Supplementary References**

1 Pinton, S. *et al.* Amyloid detection and typing yield of skin biopsy in systemic amyloidosis and polyneuropathy. *Ann Clin Transl Neurol* **10**, 2347-2359 (2023). <https://doi.org:10.1002/acn3.51924>

2 Afonine, P. V. *et al.* New tools for the analysis and validation of cryo-EM maps and atomic models. *Acta Crystallogr D Struct Biol* **74**, 814-840 (2018). <https://doi.org:10.1107/S2059798318009324>

3 Saelices, L. *et al.* Uncovering the Mechanism of Aggregation of Human Transthyretin. *J Biol Chem* **290**, 28932-28943 (2015). <https://doi.org:10.1074/jbc.M115.659912>

4 Schymkowitz, J. *et al.* The FoldX web server: an online force field. *Nucleic Acids Res* **33**, W382-388 (2005). <https://doi.org:10.1093/nar/gki387>

Uncropped gel shown in **Supplementary Figure 1b**.
