# Supplementary material for: Structure of ATTRv-F64S fibrils isolated from skin tissue of a living patient
Source: Nat Commun. 2025 Dec 16;17:781. doi: 10.1038/s41467-025-67457-2 (PMC12824241; doi:10.1038/s41467-025-67457-2)
Supplement: Supplementary file 2 — Reporting Summary [file 41467_2025_67457_MOESM2_ESM.pdf]

nature portfolio

Corresponding author(s): Andreas Boland
Giorgia Melli

Last updated by author(s): Jul 23, 2025

Reporting Summary

Nature Portfolio wishes to improve the reproducibility of the work that we publish. This form provides structure for consistency and transparency in reporting. For further information on Nature Portfolio policies, see our Editorial Policies and the Editorial Policy Checklist.

Statistics

For all statistical analyses, confirm that the following items are present in the figure legend, table legend, main text, or Methods section.

n/a	Confirmed	
☐	☒	The exact sample size (*n*) for each experimental group/condition, given as a discrete number and unit of measurement
☐	☒	A statement on whether measurements were taken from distinct samples or whether the same sample was measured repeatedly
☒	☐	The statistical test(s) used AND whether they are one- or two-sided<br>*Only common tests should be described solely by name; describe more complex techniques in the Methods section.*
☒	☐	A description of all covariates tested
☒	☐	A description of any assumptions or corrections, such as tests of normality and adjustment for multiple comparisons
☒	☐	A full description of the statistical parameters including central tendency (e.g. means) or other basic estimates (e.g. regression coefficient) AND variation (e.g. standard deviation) or associated estimates of uncertainty (e.g. confidence intervals)
☒	☐	For null hypothesis testing, the test statistic (e.g. *F*, *t*, *r*) with confidence intervals, effect sizes, degrees of freedom and *P* value noted<br>*Give P values as exact values whenever suitable.*
☒	☐	For Bayesian analysis, information on the choice of priors and Markov chain Monte Carlo settings
☒	☐	For hierarchical and complex designs, identification of the appropriate level for tests and full reporting of outcomes
☒	☐	Estimates of effect sizes (e.g. Cohen's *d*, Pearson's *r*), indicating how they were calculated

Our web collection on statistics for biologists contains articles on many of the points above.

Software and code

Policy information about availability of computer code

Data collection	Commercial software was used: EPU from Thermo Fisher Scientific for automated cryo-EM data collection.
Data analysis	Cryo-EM data acquisition was monitored by on-the-fly pre-processing software cryoSPARC (37).<br><br>Beam-induced motion correction for all movies was performed using RELION’s own implementation of the UCSF motioncor2 program (29). Contrast Transfer Function (CTF) parameters were estimated by Gctf (38). All further processing steps were carried out using RELION 4.0 (29). Fibrils were auto-picked with a binarization threshold of -6 using a modified version of the Topaz module (28).<br><br>The cryo-EM structure of ATTRv-V30M (PDB: 6SDZ) was used as an initial reference for model building. The initial model was fitted into the cryo-EM map using Chimera X (34), followed by manual and iterative building in Coot (39), before real-space refined using PHENIX (40). Model validation was performed with MolProbity (41). The FSC curve between the cryo-EM map and the atomic coordinates was calculated using Mtriage (35). Structural figures were generated in Chimera X (34).

For manuscripts utilizing custom algorithms or software that are central to the research but not yet described in published literature, software must be made available to editors and reviewers. We strongly encourage code deposition in a community repository (e.g. GitHub). See the Nature Portfolio guidelines for submitting code & software for further information.

Data

Policy information about availability of data

All manuscripts must include a data availability statement. This statement should provide the following information, where applicable:

- Accession codes, unique identifiers, or web links for publicly available datasets
- A description of any restrictions on data availability
- For clinical datasets or third party data, please ensure that the statement adheres to our policy

The EM maps of the ATTR-F64S fibril structures - extracted from ankle skin tissue - have been deposited in the Electron Microscopy Data Bank (EMDB) under the accession code EMD-52519. Protein coordinates for the ATTR-F64S fibril structure have been deposited in the Protein Data Bank (PDB) under accession code 9HYW

The mass spectrometry proteomics data have been deposited to the ProteomeXchange Consortium via the PRIDE (45) partner repository with the dataset identifier PXD065685.

Research involving human participants, their data, or biological material

Policy information about studies with human participants or human data. See also policy information about sex, gender (identity/presentation), and sexual orientation and race, ethnicity and racism.

Reporting on sex and gender	To protect the anonymity of the patient we prefer not to disclose the sex and gender.
Reporting on race, ethnicity, or other socially relevant groupings	Not relevant to this study.
Population characteristics	To protect the anonymity of the patient we prefer not to disclose the sex and gender.
Recruitment	The individual was selected based on clinical findings.
Ethics oversight	This study was performed in line with the principles of the Declaration of Helsinki and the protocol, approved by the Cantonal Ethics Committee (CETi 2895).

Note that full information on the approval of the study protocol must also be provided in the manuscript.

Field-specific reporting

Please select the one below that is the best fit for your research. If you are not sure, read the appropriate sections before making your selection.

☒ Life sciences ☐ Behavioural & social sciences ☐ Ecological, evolutionary & environmental sciences

For a reference copy of the document with all sections, see nature.com/documents/nr-reporting-summary-flat.pdf

Life sciences study design

All studies must disclose on these points even when the disclosure is negative.

Sample size	EM studies: We collected a total of 9,763 micrographs. A total number of 4,599,018 segments has been extracted, of which 12,633 segments were used for a final reconstruction. This reconstruction refined to a final resolution of 2.8 Angstrom (see Table S2). Sample sizes were estimated according to common methodology and analysis in cryo-EM studies.
Data exclusions	No data sets were excluded, unless specified in the methods section (see paragraph 'Cryo-EM image processing')
Replication	*Describe the measures taken to verify the reproducibility of the experimental findings. If all attempts at replication were successful, confirm this OR if there are any findings that were not replicated or cannot be reproduced, note this and describe why.*
Randomization	The data shown represents a single case study for a specific genetic variant, therefore randomization is not relevant to study.
Blinding	Blinding was not practical or relevant to this study. However, all analyses were automated or streamlined, and no bias was invoked.

Reporting for specific materials, systems and methods

We require information from authors about some types of materials, experimental systems and methods used in many studies. Here, indicate whether each material, system or method listed is relevant to your study. If you are not sure if a list item applies to your research, read the appropriate section before selecting a response.

Materials & experimental systems

n/a	Involved in the study	
☐	☒	Antibodies
☒	☐	Eukaryotic cell lines
☒	☐	Palaeontology and archaeology
☒	☐	Animals and other organisms
☒	☐	Clinical data
☒	☐	Dual use research of concern
☒	☐	Plants

Methods

n/a	Involved in the study	
☒	☐	ChIP-seq
☒	☐	Flow cytometry
☒	☐	MRI-based neuroimaging

Antibodies

Antibodies used	PGP9.5 (Protein Gene Product 9.5) antibody
Validation	PGP9.5 (Protein Gene Product 9.5) antibody has been validated by the group of Giorgia Melli, Institute for Translational Research, Bellinzona (ref. 23)

Plants

Seed stocks	N/A
Novel plant genotypes	N/A
Authentication	N/A
